# Suicidal ideation and attempts in patients with major depression: Sociodemographic and clinical variables

**DOI:** 10.4103/0019-5545.43059

**Published:** 2005

**Authors:** A.S. Srivastava, Rakesh Kumar

**Affiliations:** * Reader and Head; ** Senior Resident

**Keywords:** Suicidal ideation, suicidal attempt, sociodemographic variables, Hamilton Rating Scale for Depression (HAM-D), major depression

## Abstract

**Background:**

There is a high risk of suicidal attempt in patients with depression. This risk varies according to the sociodemographic status and clinical presentation.

**Aim:**

To assess the prevalence of suicidal ideation and attempt in patients with major depressive disorder, and to find the correlation between the two.

**Methods:**

Sixty patients with major depressive disorder having suicidal ideation were recruited in the study. Of these, 10 had a history of suicidal attempt made in the past or current episode. Sociodemographic details were evaluated and the Hamilton Rating Scale for Depression (HAM-D) was administered.

**Results:**

Analysis of the data showed that the incidence of suicidal attempt was 16.6% in patients with suicidal ideation. Statistically, a higher risk of suicidal attempt was found in individuals <30 years of age. Single men, married women and students were more likely to attempt suicide; higher education was also a vulnerability factor. Attempters scored significantly higher in severity of suicidal ideation, agitation and paranoid symptoms whereas among non-attempters, hypochondriasis and general somatic symptoms were more often present.

**Conclusion:**

All patients expressing suicidal ideation do not attempt suicide. Young patients with depression, especially unmarried men, married women and students, having severe suicidal ideation with agitation or paranoid symptoms are more likely to attempt suicide.

## INTRODUCTION

Psychiatric patients who attempt suicide have greater suicidal ideation compared with patients who do not attempt suicide.^1^ Suicidal ideation is present and appears to be a precondition for suicide attempts in patients with major depressive disorder. Sokero *et al*^2^ found that among 15% of those who had attempted suicide, the majority (95%) had suicidal ideation. Age, sex and education failed to distinguish between suicide attempters and non-attempters.^3^ Srivastava *et al*^4^ found a positive correlation between suicide attempt and the severity of depression, male sex, being married, employed and ≤35 years of age. Higher levels of suicidal ideation, aggression and hostility were reported in subjects who attempted suicide.^5^

The present study was carried out to see if there was a correlation between suicidal ideation and suicidal attempt in patients with major depressive disorder. The sociodemographic and clinical variables of the patients were also analysed.

## METHODS

The study sample consisted of 60 patients (31 men and 29 women) attending the Psychiatry Outpatient Department of the Institute of Medical Sciences, Banaras Hindu Universty, Varanasi from June to November 2004. Patients between 16 and 60 years of age who presented for treatment of a major depressive episode and had suicidal ideation were included in the study. The mean age of the sample was 31.38±11.59 years. The exclusion criteria were current substance or alcohol abuse, neurological illness, active medical conditions, history of use of any psychotropic medication and other psychiatric illnesses. All patients underwent a physical examination and routine blood tests were done.

A semi-structured proforma was used to record the sociodemographic variables, and psychiatric and medical history of the patients. They were clinically assessed for depression. The severity of the major depressive episode was measured objectively using the Hamilton Rating Scale for Depression (HAM-D).^6^ Diagnosis of the current depressive episode was based on the DSM-IV criteria.^7^ Chi-square test was applied to test the association between the sociodemographic variables. Mean and standard deviation of different symptom variables of HAM-D were calculated. To test the statistical difference between the two groups, independent sample *t* test was applied. A value of p<0.05 was considered statistically significant whereas p>0.05 was considered statistically non-significant.

## RESULTS

### Sociodemographic profile

Table 1 gives the sociodemographic profile of the patients. Among the 60 patients, 10 had attempted suicide (16.6%). However, no one more than 30 years of age had attempted suicide.

**Table 1:** Sociodemographic profile of the study population*

Variable	Attempters n=10 (16.6%)	Non-attempters n=50 (83.4%)	χ^2^	df	p
Age (years)					
Up to 30 (*n*=36)	10 (27.7)	26 (72.3)	6.13	1	0.013
Above 30 (*n*=24)	0	24 (100)			
Sex					
Male (*n*=31)	6 (19.4)	25 (80.6)	0.05	1	NS
Female (*n*=29)	4 (13.8)	25 (86.2)			
Location					
Rural (*n*=34)	4 (11.8)	30 (88.2)	0.67	1	NS
Urban (*n*=26)	6 (23)	20 (77)			
Marital status					
Married (*n*=44)	7 (15.9)	37 (84.1)	0.02	1	NS
Single (*n*=16)	3 (18.75)	13 (81.25)			
Education					
Below X (*n*=21)	1 (4.8)	20 (95.2)	2.11	1	NS
Above X (*n*=39)	9 (23)	30 (77)			
Occupation					
Housewife (*n*=24)	4 (16.7)	20 (83.3)	1.85	2	NS
Student (*n*=15)	4 (26.6)	11 (73.4)			
Others (*n*=21)	2 (9.5)	19 (90.5)			

* All the patients expressed suicidal ideation

Values in parentheses are percentages.

Thirty-four patient were from rural areas and 26 were from urban areas. More patients from urban areas attempted suicide compared with those from rural areas (23% vs. 11.8%). Forty-four patients were married (73.3%); among them, 7 had attempted suicide (15.9%). Of the 16 unmarried patients (26.6%), 3 had attempted suicide (18.7%) and all of them were men.

Thirty-nine patients (65%) had studied up to or higher than class 10. Among these, 9 patients had attempted suicide (23%). On the other hand, only 1 patient (4.8%) had attempted suicide among those who were educated below the level of class 10 (21 [35%]).

Twenty-four patients were housewives (40%), 15 were students (25%) and 21 were either self-employed, government employees, farmers or unemployed (35%). Students were the most vulnerable group for attempted suicide (26.6%), followed by housewives (16.7%) and those in the ‘others’ category (9.5%).

Table 2 gives the psychiatric history of attempters and non-attempters.

**Table 2:** Psychiatric history of attempters and non-attempters

Variable	Non-attempters	Attempters
Family history of psychiatric illness	8	2
Past history of psychiatric illness	13	4
History of past suicidal attempt	–	3
History of stressor	27	6

### Clinical characteristics

Table 3 compares the clinical characteristics of attempters with non-attempters based on the HAM-D. The mean and standard deviation (SD) of symptom variables in HAM-D were calculated for the two groups. The two groups were compared using the *t* test. The intensity of suicidal thoughts, agitation and paranoid symptoms were more in attempters, whereas general somatic symptoms and hypochondriasis were more in non-attempters. The difference between attempters and non-attempters on other variables was statistically not significant.

**Table 3:** Clinical characteristics of attempters versus non-attempters based on the Hamilton Rating Scale for Depression (HAM-D)

	Non-attempters Attempters (*n*=50)		Attempters (*n*=10)		Analysis	
			
Symptom variables of HAM-D	Mean	SD	Mean	SD	*t*	p
Depressed mood	2.80	0.57	2.40	0.84	1.86	>0.05
Feeling of guilt	0.64	0.85	1.10	1.10	1.48	>0.05
Intensity of suicidal ideation	2.98	0.14	3.60	0.52	7.41	<0.001 S
Insomnia						
Early	1.78	0.42	1.60	0.70	1.10	>0.05
Middle	0.52	0.51	0.70	0.61	0.97	>0.05
Late	0.28	0.64	0.20	0.63	0.36	>0.05
Work and activities	3.02	0.74	2.80	0.63	0.87	>0.05
Retardation	1.30	0.73	1.00	1.15	1.06	>0.05
Agitation	0.22	0.58	0.90	1.19	2.75	<0.01 S
Anxiety						
Psychic	1.68	0.58	2.00	0.67	1.54	>0.05
Somatic	1.58	0.61	1.20	0.79	1.71	>0.05
Somatic symptoms						
G.I.	1.10	0.36	1.10	0.74	0.00	>0.05
General	1.70	0.46	1.10	0.88	3.16	<0.01 S
Genital symptoms	0.88	0.52	0.70	0.67	0.95	>0.05
Hypochondriasis	0.78	0.88	0.10	0.32	2.38	<0.05 S
Loss of weight	0.86	0.73	0.90	0.74	0.16	<0.05
Insight	0.68	0.51	0.90	0.57	1.22	<0.05
Diurnal variation	0.32	0.51	0.30	0.48	0.11	>0.05
Depersonalization and derealization	0.02	0.14	0.10	0.32	1.28	>0.05
Paranoid symptoms	0.02	0.14	0.40	0.52	4.54	<0.001 S
Obsessional symptoms	0.08	0.27	0.20	0.63	0.98	>0.05
Total	23.24	2.97	23.30	4.74	0.05	>0.05

## DISCUSSION

In this study, the incidence of suicidal attempt was 16.6% in patients with a major depressive episode and suicidal ideation, which is comparable to that reported by Sokero *et al*^2^ (15%).

All the attempters were <30 years of age. This is comparable to the study by Narang *et al*^8^ where 73% of the attempters were <30 years of age. Kessler *et al*^9^ reported that age <25 years is a significant risk factor for suicidal attempt among those with suicidal ideation. However, to get a more comprehensive trend in this area, a study with a much larger sample should be conducted.

No significant difference was found between male and female attempters, which is in concurrence with the findings of other Indian studies.^10^ However, Kessler *et al*^9^ found that more women were at risk for suicidal attempt and suicidal ideation.

In this study, the representation from rural areas was higher compared with urban areas, but a higher number of patients from the urban background attempted suicide, which is comparable to the findings of Narang *et al*^8^

Another finding of this study was that a higher number of single men and married women attempted suicide compared with married men and unmarried women. This finding was in concurrence with that reported in previous studies.^8^,^11^–^13^

It was observed that patients in the more educated group were more likely to attempt suicide. However, Kessler *et al*^9^ found that poorly educated subjects were more vulnerable to suicidal attempts. Among attempters, students and housewives outnumbered the ‘others’ group, which is in concurrence with the study by Narang *et al*^8^

Malone *et al*^5^ reported a higher level of suicidal ideation, aggression, hostility and impulsivity in subjects who attempted suicide. This was also observed in the present study where attempters had a higher level of suicidal ideation and agitation. Malone *et al.,* however, did not find any correlation between suicidal attempt and paranoid symptoms. Srivastava *et al*^4^ also reported a low positive relationship between severity of depression and suicidal intent. Botswick *et al*^14^ also reported that impulsivity, aggression and psychosis increased in attempters. Roose *et al*^15^ concluded that delusional, depressed patients were five times more likely to commit suicide than non-delusional ones.

## CONCLUSION

All the patients who express suicidal ideation do not attempt suicide. Suicidal attempts were reported in only 16.6% of the patients with major depressive episodes having suicidal ideation. A significantly higher number of patients in the age group of <30 years attempted suicide. Intensity of suicidal thoughts, agitation and paranoid symptoms were more in attempters, whereas general somatic symptoms and hypochondriasis were more in non-attempters. Young patients with depression, especially unmarried men, housewives, students and those with class 10 or higher education, having severe suicidal ideation with agitation or paranoid symptoms are more prone to attempt suicide.
